# Metabolically healthy versus unhealthy obese phenotypes in relation to hypertension incidence; a prospective cohort study

**DOI:** 10.1186/s12872-022-02553-5

**Published:** 2022-03-14

**Authors:** Behrooz Hamzeh, Yahya Pasdar, Shima Moradi, Mitra Darbandi, Negin Rahmani, Ebrahim Shakiba, Farid Najafi

**Affiliations:** 1 Health Education and Promotion, Research Center for Environmental Determinants of Health (RCEDH), Health Institute, Kermanshah University of Medical Sciences, Kermanshah, Iran; 2 Department of Nutrition Sciences, Research Center for Environmental Determinants of Health (RCEDH), Kermanshah University of Medical Sciences, Kermanshah, Iran; 3 Department of Nutrition Sciences, Research Center for Environmental Determinants of Health (RCEDH), Health Institute, Kermanshah University of Medical Sciences, Kermanshah, Iran; 4 Research Center for Environmental Determinants of Health (RCEDH), Health Institute, Kermanshah University of Medical Sciences, Kermanshah, Iran; 5 Julius Maximillian University of Wuerzburg, Wuerzburg, Germany; 6 Social Development and Health Promotion Research Center, Kermanshah University of Medical Sciences, Kermanshah, Iran; 7 Epidemiology, School of Public Health, Communing Developmental and Health Promotion Research Center, Kermanshah University of Medical Sciences, Kermanshah, Iran

**Keywords:** Metabolically unhealthy obesity, Metabolically healthy obesity, Hypertension, Incidence, PERSIAN

## Abstract

**Background:**

Although obesity increases the risk of hypertension, the effect of obesity based on metabolic status on the incidence of hypertension is not known. This study aimed to determine the association between obesity phenotypes including metabolically unhealthy obesity (MUO) and metabolically healthy obesity (MHO) and the risk of hypertension incidence.

**Methods:**

We conducted a prospective cohort study on 6747 adults aged 35–65 from Ravansar non-communicable diseases (RaNCD) study. Obesity was defined as body mass index above 30 kg/m^2^ and metabolically unhealthy was considered at least two metabolic disorders based on the International Diabetes Federation criteria. Obesity phenotypes were categorized into four groups including MUO, MHO, metabolically unhealthy non obesity (MUNO), and metabolically healthy non obesity (MHNO). Cox proportional hazards regression models were applied to analyze associations with hypertension incidence.

**Results:**

The MHO (HR: 1.37; 95% CI: 1.03–1.86) and MUO phenotypes (HR: 2.44; 95% CI: 1.81–3.29) were associated with higher hypertension risk compared to MHNO. In addition, MUNO phenotype was significantly associated with risk of hypertension incidence (HR: 1.65; 95% CI: 1.29–2.14).

**Conclusions:**

Both metabolically healthy and unhealthy obesity increased the risk of hypertension incidence. However, the increase in metabolically unhealthy phenotype was higher.

## Background

Hypertension is one of the strongest modifiable risk factors for cardiovascular disease (CVD) with its prevalence increasing especially in low- and middle-income countries [1, 2]. Reports indicate that a quarter of men and a fifth of women have hypertension, and hypertension is responsible for approximately 45% of deaths from CVDs [3, 4]. Results of systematic review and meta-analysis on 42 Iranian studies showed that hypertension affects 22% of 402,282 subjects included in this analysis [5].

Many factors contribute to hypertension including sedentary lifestyle, kidney disease, diabetes, obesity, high salt intake, and processed foods [6, 7]. Among these factors, obesity contributes also to the development of CVDs, type 2 diabetes, cancer, and inflammatory diseases [8–11]. Evidence suggests that obesity, with its pro-inflammatory effects and oxidative stress, can cause insulin resistance, dyslipidemia, and other metabolic disorders. This is called metabolically unhealthy obesity (MUO) [12, 13]. Additionally, metabolically unhealthy non obesity (MUNO) phenotypes are at risk of type 2 diabetes, CVDs, fatty liver, and higher mortality [13, 14]. Nevertheless, some people with obesity have metabolically healthy status and they are said to have metabolically healthy obesity (MHO) phenotype [12]. Most studies focus on people with unhealthy metabolic status, and studies on MHO phenotype are limited. It is clear that MHO phenotype and its health consequences are not well understood [2, 15]. Evidence suggests that these people are at less risk for some of the mentioned diseases compared with MUO, but still have a higher risk of these diseases compared with people with normal weight. In general, MHO phenotype should not be considered a safe condition that does not require treatment for obesity [15].

Reports indicate that obesity is associated with the risk of developing hypertension. Since no study has examined the types of obesity phenotypes based on the metabolic status of individuals yet, the present study was conducted with the aim of identifying the association between metabolically healthy versus unhealthy obese phenotypes and the risk of hypertension incidence in the Ravansar non-communicable diseases (RaNCD) cohort study.

## Methods

### Study design and setting

We conducted a prospective cohort study using data from the RaNCD cohort study. The RaNCD study is the first cohort study on the Kurdish population in Iran which started in October 2014. The subjects are 35–65 years old and live in Ravansar city, Kermanshah province, Western-Iran. The RaNCD cohort study is a part of the PERSIAN (Prospective Epidemiological Research Studies in Iran) mega cohort study that was approved by the Ethics Committees in the Ministry of Health and Medical Education, the Digestive Diseases Research Institute, Tehran University of Medical Sciences, Iran. The details of this study were described in previous studies [16, 17]. In this study, we included all the recruitment phase participants who were surveyed from October 2014 to January 2017 and followed up until January 2021 (n = 4764 men and 5283 women). The RaNCD cohort study was approved by the Ethics Committee of Kermanshah University of Medical Sciences (No: IR.KUMS.REC.1400.268).

### Participants

Among RaNCD participants, 3300 were not included in the study for the following reasons: (1) Diseases such as CVDs (n = 1709), type 2 diabetes (n = 870), hypertension (n = 1579), cancer (n = 83), and thyroid diseases (n = 763); (2) pregnancy (n = 138); and (3) energy intake less than 800 kcal/day or more than 4200 kcal/day (n = 737). After excluding participants with missing data, 6747 participants were included into this study. Among them, 393 participants were identified as new cases of hypertension incident after follow-up, and the rest were considered as the sub-cohort group.

### Measurements

The current study obtained demographic data including age, sex, smoking status, and physical activity as well as medical history, medication, anthropometric indices, blood pressure, and biochemical analysis.

### Anthropometry

The height of all the participants was measured by the automatic stadiometer BSM 370 (Biospace Co., Seoul, Korea) with a precision of 0.1 cm in standing position without shoes. InBody 770 device (Inbody Co, Seoul, Korea) was applied to measure the weight and body fat mass (BFM) of participants with the least clothing and without shoes. To determine obesity, body mass index (BMI) was calculated by dividing the weight in kilograms by height in meters squared. BMI above 30 kg/m^2^ was considered as obesity. Waist circumference (WC) was measured using a non-stretched and flexible tape at the level of the iliac crest in standing position [18].

### Blood pressure

In RaNCD cohort study, conventional sphygmomanometry and auscultation of Korotkoff sounds was used to measure systolic and diastolic blood pressure (SBP and DBP) in sitting position after at least 4–5 min of rest. The blood pressure measurement was conducted twice with a 10 min interval and the average was calculated and reported as the final blood pressure [16].

### Biochemical analysis

25 cc blood samples were collected from all RaNCD participants. The serum and whole blood samples were subdivided and stored at − 80 °C at the RaNCD cohort laboratory until analysis. Serum fasting blood sugar (FBS) was measured by glucose oxidase method. Total cholesterol (TC), high-density lipoprotein (HDL), triglyceride (TG) and low-density lipoprotein (LDL) concentrations were measured by enzymatic kits (Pars Azmun, Iran) [16].

### Obesity phenotypes

We defined MUO by the presence of BMI > 30 kg/m^2^ and at least two metabolic disorders according to the International Diabetes Federation (IDF) statement [19] as follow: HDL < 40 mg/dl in men and < 50 mg/dl in women, TG > 150 mg/dl, SBP > 130 mmHg or DBP > 80 mmHg or receiving antihypertensive medication, and FBS > 100 mg/dl or receiving medication for diabetes. MHO was defined as BMI > 30 kg/m^2^ and having at most one metabolic disorder mentioned above. MUNO phenotype was defined by the presence of BMI < 30 kg/m^2^ and at least two of the above-mentioned metabolic disorders. Finally, MHNO participants were defined as healthy participants without obesity and metabolic disorder or having at most one metabolic disorder.

### Outcome measurement of hypertension incidence

In the RaNCD study, participants are monitored for blood pressure each year and their systolic and diastolic blood pressure are measured. The medications and medical history of hypertension during the follow-up period are also assessed by the RaNCD physician. Hypertension was defined by SBP/DBP ≥ 140/90 mmHg and/or using anti-hypertensive medications in the time interval between baseline (first phase of Ravansar cohort which has been conducted since 2014) and hypertension diagnosis (from 2015 to 2021). The overall duration of the follow-up was 32,596 person/year.

### Statistical analysis

Statistical analysis was performed using Stata, version 14 (Stata Corp, College Station, TX). Mean ± standard deviation (SD) and frequency percentage were used to report baseline characteristics of studied participants. To compare the results of baseline characteristics among different obesity phenotypes, one-way analysis of variance (ANOVA) was used for continuous variables, and a Chi-square test was used for categorical variables. The number of degrees of freedom (df) used to calculate *P* values was 3.

Cox proportional hazards regression model was used to calculate hazard ratios (HRs) stratified by obesity phenotypes, with hypertension as the event and the time interval between baseline (first phase of RaNCD cohort) and hypertension diagnosis as the time covariate. This regression model was applied in the previous studies to determine hypertension incidence [20–23]. The model was adjusted for confounding variables including age, sex, physical activity, smoking and energy intake. HR was reported with 95% confidence interval (CI).

## Results

A total of 6747 participants were analyzed in this study from which 6354 participants were part of the sub-cohort and the rest (n = 393) were identified as new cases of hypertension. The percentage of hypertension new cases is demonstrated based on obesity phenotypes (Table 1).

**Table 1:** Baseline characteristics of studied participants

Variables	Total (n = 6747)	MHNO (n = 3965)	MHO (n = 1036)	MUNO (n = 1204)	MUO (n = 542)	P**
Age (year)	45.77 ± 7.76*	45.67 ± 7.97	44.99 ± 7.04	46.76 ± 7.88	45.86 ± 7.02	< 0.001
Weight (kg)	71.87 ± 13.42	66.49 ± 11.02	84.54 ± 10.83	71.76 ± 10.11	87.12 ± 12.25	< 0.001
WC (cm)	96.26 ± 10.36	92.03 ± 8.64	106.89 ± 8.23	95.94 ± 7.01	107.54 ± 8.81	< 0.001
BMI (kg/m^2^)	27.01 ± 4.67	24.73 ± 3.34	33.11 ± 2.93	26.45 ± 2.45	33.33 ± 3.28	< 0.001
BFM (kg)	24.27 ± 9.41	19.76 ± 6.61	36.38 ± 6.78	22.41 ± 5.25	36.24 ± 7.61	< 0.001
SBP (mmHg)	103.55 ± 12.42	101.62 ± 11.83	104.10 ± 11.31	107.16 ± 13.07	108.59 ± 13.83	< 0.001
DBP (mmHg)	67.44 ± 7.82	66.37 ± 7.36	67.76 ± 7.51	69.44 ± 8.28	70.18 ± 8.95	< 0.001
FBS (mg/dl)	89.92 ± 9.49	87.91 ± 8.03	88.96 ± 8.08	94.36 ± 11.29	96.58 ± 11.04	< 0.001
TC (mg/dl)	184.01 ± 36.79	180.03 ± 37.31	186.74 ± 33.72	190.40 ± 36.80	193.68 ± 34.36	< 0.001
TG (mg/dl)	130.01 ± 73.75	101.75 ± 46.35	114.06 ± 47.34	205.82 ± 87.54	198.37 ± 83.06	< 0.001
HDL (mg/dl)	46.83 ± 11.41	49.67 ± 11.21	49.82 ± 10.55	38.04 ± 7.45	39.85 ± 8.31	< 0.001
LDL (mg/dl)	101.26 ± 24.90	98.66 ± 25.16	102.64 ± 22.76	105.89 ± 25.49	107.33 ± 22.79	< 0.001
PA (MET hour/day)	41.08 ± 8.15	41.90 ± 8.78	39.71 ± 6.19	40.37 ± 7.78	39.32 ± 6.55	< 0.001
Current smoking (%)	11.9	20.7	9.4	23.1	14.1	< 0.001
Hypertension incidence	5.79	4.4	6.2	7.7	10.9	< 0.001

*MHNO* metabolically healthy non-obese, *MHO* metabolically healthy obese, *MUNO* metabolically unhealthy non-obese, *MUO* metabolically unhealthy obese, *WC* waist circumference, *BMI* body mass index, *BFM* body fat mass, *SBP* systolic blood pressure, *DBP* diastolic blood pressure, *FBS* fasting blood sugar, *TC* total cholesterol, *TG* triglyceride, *HDL* high density lipoprotein, *LDL* low density lipoprotein, *PA* physical activity

*Mean ± SD

**P values were obtained one-way ANOVA and Chi square

The prevalence of MHO, MUNO, and MUO were 15.3, 17.4, and 8.03%; respectively. The mean physical activity in MHNO was significantly higher than the other three obesity phenotypes (MHO, MUNO, and MUO) in both men and women. Table 2 presents baseline characteristics of studied participants based on the obesity phenotypes.

**Table 2:** Baseline characteristics of studied participants based on the different types of obesity phenotypes

Variables	Men (n = 3217)				P**	Women (n = 3530)				P**
	MHNO (n = 2094)	MHO (n = 246)	MUNO (n = 692)	MUO (n = 185)		MHNO (n = 1871)	MHO (n = 790)	MUNO (n = 512)	MUO (n = 357)	
Age (year)	45.95 ± 7.88*	45.13 ± 7.09	45.96 ± 7.40	44.98 ± 7.01	0.174	45.35 ± 8.06	44.94 ± 7.03	47.84 ± 8.37	46.31 ± 6.99	< 0.001
Weight (kg)	71.01 ± 10.74	94.50 ± 9.53	76.69 ± 8.93	95.74 ± 10.33	< 0.001	61.44 ± 8.94	81.44 ± 9.22	65.11 ± 7.47	82.65 ± 10.70	< 0.001
WC (cm)	92.49 ± 8.30	107.53 ± 7.70	96.14 ± 6.87	106.97 ± 7.71	< 0.001	91.51 ± 8.97	106.68 ± 8.39	95.66 ± 7.20	107.83 ± 9.32	< 0.001
BMI (kg/m^2^)	24.36 ± 3.40	32.42 ± 2.30	26.26 ± 2.52	32.37 ± 2.29	< 0.001	25.15 ± 3.22	33.32 ± 3.07	26.70 ± 2.34	33.82 ± 3.60	< 0.001
BFM (kg)	17.74 ± 6.24	33.77 ± 6.69	20.91 ± 5.01	33.54 ± 7.17	< 0.001	22.31 ± 6.18	37.32 ± 6.58	24.71 ± 4.77	37.90 ± 7.41	< 0.001
SBP (mmHg)	103.59 ± 11.53	108.20 ± 11.10	107.77 ± 12.22	110.03 ± 12.87	< 0.001	99.41 ± 11.77	102.83 ± 11.08	106.33 ± 14.11	107.84 ± 14.26	< 0.001
DBP (mmHg)	67.31 ± 7.50	70.30 ± 7.65	69.98 ± 8.00	71.25 ± 8.77	< 0.001	65.32 ± 7.05	66.97 ± 7.29	68.72 ± 8.60	69.62 ± 9.00	< 0.001
FBS (mg/dl)	88.32 ± 8.14	89.74 ± 8.57	93.71 ± 10.90	95.53 ± 9.94	< 0.001	87.45 ± 7.88	88.72 ± 7.91	95.25 ± 11.74	97.12 ± 11.55	< 0.001
TC (mg/dl)	178.53 ± 36.02	187.56 ± 35.36	185.02 ± 33.58	188.54 ± 30.04	< 0.001	181.72 ± 38.64	186.49 ± 33.22	197.68 ± 39.64	196.34 ± 36.15	< 0.001
TG (mg/dl)	108.41 ± 51.12	134.62 ± 67.99	216.65 ± 89.80	220.14 ± 86.05	< 0.001	94.28 ± 39.00	107.65 ± 36.49	191.19 ± 82.24	187.09 ± 79.27	< 0.001
HDL (mg/dl)	46.37 ± 9.98	44.71 ± 8.98	35.24 ± 5.92	35.29 ± 5.74	< 0.001	53.39 ± 11.35	51.41 ± 10.50	41.82 ± 7.64	42.22 ± 8.46	< 0.001
LDL (mg/dl)	99.23 ± 24.50	106.41 ± 23.50	103.03 ± 22.89	105.91 ± 20.73	< 0.001	98.03 ± 25.87	101.46 ± 22.42	109.77 ± 28.19	108.08 ± 23.78	< 0.001
PA (MET hour/day)	43.70 ± 10.87	41.59 ± 9.87	41.31 ± 9.59	40.09 ± 9.78	< 0.001	39.89 ± 4.84	39.12 ± 4.30	39.10 ± 3.92	38.93 ± 3.91	< 0.001
Current smoking (%)	35.9	30.7	34.8	35.3	0.462	3.7	2.8	7.1	3.1	0.001
Hypertension incidence	4	6.5	5.1	8.1	0.029	4.9	6.1	11.3	12.3	< 0.001

*MHNO* metabolically healthy non-obese, *MHO* metabolically healthy obese, *MUNO* metabolically unhealthy non-obese, *MUO* metabolically unhealthy obese, *WC* waist circumference, *BMI* body mass index, *BFM* body fat mass, *SBP* systolic blood pressure, *DBP* diastolic blood pressure, *FBS* fasting blood sugar, *TC* total cholesterol, *TG* triglyceride, *HDL* high density lipoprotein, *LDL* low density lipoprotein, *PA* physical activity

*Mean ± SD

**P values were obtained one-way ANOVA and Chi square

The risk of incident hypertension increased in MHO phenotype compared to MHNO (HR: 1.41; 95% CI: 1.05–1.88) in model I, which remained significant after adjustment for age, sex, physical activity and smoking (HR: 1.37; 95% CI: 1.03–1.86). The risk of incident hypertension increased in MUO phenotype compared to MHNO (HR: 2.44; 95% CI: 1.81–3.29) after adjust sex and age, which remained significant after adjustment for age, sex, physical activity and smoking (HR: 2.40; 95% CI: 1.77, 3.26) (Table 3).

**Table 3:** Hazard ratio of incident hypertension according to obesity phenotypes

Obesity phenotypes	N	% (N) of cases	Hazard ratio (95% CI)		
			Model I	Model II	Model III
MHNO	3965	4.4 (175)	Ref.	Ref.	Ref.
MHO	1036	6.2 (64)	1.41 (1.05, 1.88)	1.37 (1.02, 1.83)	1.37 (1.03, 1.86)
MUNO*	1204	7.7 (93)	1.68 (1.31, 2.16)	1.64 (1.27, 2.11)	1.65 (1.29, 2.14)
MUO	542	10.9 (59)	2.44 (1.81, 3.29)	2.36 (1.75, 3.20)	2.40 (1.77, 3.26)

Model I: adjusted for age and sex; Model II: adjusted for age, sex and physical activity; Model III: adjusted for age, sex, physical activity and smoking

*MHNO* metabolically healthy non-obese, *MHO* metabolically healthy obese, *MUNO* metabolically unhealthy non-obese, *MUO* metabolically unhealthy obese

**Fig. 1:**
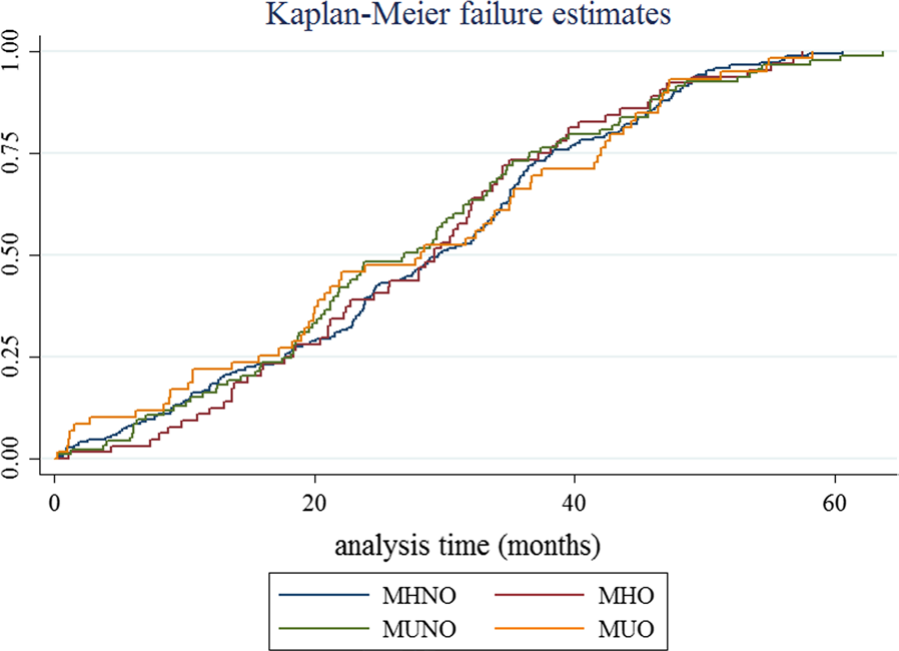
Cumulative hazard curves for the incidence of hypertension in over time according to obesity phenotypes

In addition, risk of hypertension significantly increased in MUNO phenotype compared to MHNO in all adjusted models (HR: 1.65; 95% CI: 1.29–2.14). The cumulative hazard curves show that the incidence of hypertension has increased by approximately 7% in MUO phenotype over 5 year; and this increase was more than other phenotypes over time (Fig. 1).

## Discussion

Our results show that both MHO and MUO phenotypes increase the risk of hypertension compared to MHNO phenotype. Furthermore, MUNO phenotype was associated with a higher risk of hypertension incidence compared to MHNO phenotype. Overall, the MUO phenotype increased the risk of hypertension incidence more than the other phenotypes in the follow-up time of the study. The obesity epidemic is growing and increases the risk of chronic non-communicable diseases leading to increased health system costs [24]. Epidemiological studies highlight the persistent link between obesity and hypertension, and the presence of obesity increases the risk of developing hypertension [7, 25, 26]. Since there are different phenotypes of obesity based on the metabolic status, we examined the association between obesity phenotypes and the risk of hypertension incidence.

The result of Whitehall II cohort study by Hinnouho et al. [27] on 5269 participants indicated that both obesity phenotypes, MHO and MUO lead to increased risk of mortality after seventeen years follow-up. Another prospective study by Fingeret et al. [28] did not find any difference between MHO and MUO in hypertension incidence after 10.9 years of follow-up (odds ratio (OR): 1.3, CI 95%: 0.8–2.09). Yuan et al. [29] showed that MHO has no association with the development of arterial stiffness (OR: 0.99; CI 95%: 0.61–1.6), while MUO and MUNO phenotypes lead to significantly progressed arterial stiffness (OR: 4.56; CI 95%: 2.60–8) and (OR: 5.05; CI 95%: 3.12–8.19), respectively. On the other hand, some studies by Hashimoto et al. [30] and Gilardini et al. [31] did not see any association between MHO and the risk of renal failure, prediabetes, diabetes and CVDs. In addition, Zhang et al. reported that none of the phenotypes were associated with an increased risk of left ventricular hypertrophy (MHO: OR: 0.845; CI 95%: 0.239–2.987; MUNO: OR: 0.567; CI 95%: 0.316–1.018; MUO: OR: 0.632; CI 95%: 0.342–1.166) [32]. Another study by Chaffin et al. [33] showed that MHO was not associated with incident CVD. In these studies, it has been interpreted that the reason for the lack of connection between MHO phenotype and the mentioned diseases is the favorable metabolic status. Furthermore, abdominal obesity has been considered more important in causing these chronic non-communicable diseases than overall obesity.

In the current study, we observed that MUO and MUNO increase the risk of hypertension incidence more than MHO. In addition, BFM and WC of the participants were higher in all three groups compared with the MHNO phenotype. Obesity, especially the excess visceral fat distribution, increases inflammatory cytokines and endothelial disorders in which several mechanisms contribute to hypertension [24, 34, 35]. Excess adipose tissue increases the production of pro-inflammatory factors such as leptin, tumor necrosis factor-α, interleukin-6, and resistin which contribute to the development of various metabolic diseases [36]. High calorie intake and increase in adipocytes stimulate α and β adrenergic receptors, thereby increasing the activity of the sympathetic nervous system [37]. Obesity activates the renin-angiotensin system and the sympathetic nervous system, which leads to increased sodium reabsorption and arterial blood pressure [38, 39]. On the other hand, increasing adipose tissue leads to decreased adiponectin production and increased insulin resistance [40, 41]. Therefore, chronic hyperinsulinemia in obese people causes vasoconstriction and also increases urinary sodium reabsorption and is involved in the pathogenesis of hypertension [42]. In addition, increased circulating leptin levels in response to increased adipose tissue lead to impaired nitric oxide synthesis and ultimately vascular endothelial dysfunction [24]. In summary, the increased production of adipose tissue in obesity causes the production of pro-inflammatory cytokines which play an important role in the pathogenesis of hypertension by disrupting the metabolic status.

### Strengths and limitations

The present prospective study followed up the Kurdish population for the first time and examined the types of obesity based on metabolic status and the risk of hypertension incidence. In this study, we applied appropriate exclusion criteria, such as people who did not have normal calorie intake. However, this study had its limitations. First, the follow-up period seems to have been short. Second, the hypertension incidence was small for the study groups, and we could not assess the relationship between hypertension incidence and obesity phenotypes based on the sex, although it was adjusted for sex.

## Conclusion

In conclusion, the present study showed that both MHO and MUO phenotypes lead to an increase in hypertension incidence compared to MHNO phenotype. In addition, MUNO phenotype can also increase the hypertension incidence. However, MUO and MUNO phenotypes increase the risk of hypertension incidence more than MHO and MHNO phenotype. To prevent hypertension, maintaining normal body weight and controlling central obesity as well as visceral fat is highly recommended.

## Data Availability

Data will be available upon request from the corresponding author.

## References

[1] Mills KT, Stefanescu A, He J (2020). The global epidemiology of hypertension. Nat Rev Nephrol.

[2] Fisher ND, Curfman G (2018). Hypertension—a public health challenge of global proportions. JAMA.

[3] Zeng Z, Chen J, Xiao C, Chen W (2020). A global view on prevalence of hypertension and human develop index. Ann Global Health..

[4] Egan BM, Kjeldsen SE, Grassi G, Esler M, Mancia G (2019). The global burden of hypertension exceeds 1.4 billion people: should a systolic blood pressure target below 130 become the universal standard?. J Hypertens.

[5] Mirzaei M, Moayedallaie S, Jabbari L, Mohammadi M (2016). Prevalence of hypertension in Iran 1980–2012: a systematic review. J Tehran Heart Cent.

[6] Eze II, Mbachu CO, Azuogu BN, Ossai E, Unah AI, Akamike IC (2021). Effect of on-site behavioural modification intervention on lifestyle risk factors of hypertension among adult market traders in Abakaliki, Nigeria. Int J Health Promot Edu.

[7] Ostchega Y, Zhang G, Hughes JP, Nwankwo T (2018). Factors associated with hypertension control in US adults using 2017 ACC/AHA guidelines: National Health and Nutrition Examination Survey 1999–2016. Am J Hypertens.

[8] Avgerinos KI, Spyrou N, Mantzoros CS, Dalamaga M (2019). Obesity and cancer risk: emerging biological mechanisms and perspectives. Metabolism.

[9] Koliaki C, Liatis S, Kokkinos A (2019). Obesity and cardiovascular disease: revisiting an old relationship. Metabolism.

[10] Trends in obesity and diabetes across Africa from 1980 to 2014: an analysis of pooled population-based studies. Int J Epidemiol. 2017;46(5):1421–32. 10.1093/ije/dyx078 PMC5837192 28582528

[11] Darbandi M, Pasdar Y, Moradi S, Mohamed HJJ, Hamzeh B, Salimi Y (2020). Discriminatory capacity of anthropometric indices for cardiovascular disease in adults: a systematic review and meta-analysis. Prev Chronic Dis.

[12] Cӑtoi AF, Pârvu AE, Andreicuț AD, Mironiuc A, Crӑciun A, Cӑtoi C (2018). Metabolically healthy versus unhealthy morbidly obese: chronic inflammation, nitro-oxidative stress, and insulin resistance. Nutrients.

[13] Kim Y, Chang Y, Cho YK, Ahn J, Shin H, Ryu S (2019). Metabolically healthy versus unhealthy obesity and risk of fibrosis progression in non-alcoholic fatty liver disease. Liver Int.

[14] Hsu ARC, Ames SL, Xie B, Peterson DV, Garcia L, Going SB (2020). Incidence of diabetes according to metabolically healthy or unhealthy normal weight or overweight/obesity in postmenopausal women: the Women's Health Initiative. Menopause.

[15] Blüher M (2020). Metabolically healthy obesity. Endocr Rev.

[16] Pasdar Y, Najafi F, Moradinazar M, Shakiba E, Karim H, Hamzeh B (2019). Cohort profile: Ravansar non-communicable disease cohort study: the first cohort study in a Kurdish population. Int J Epidemiol.

[17] Poustchi H, Eghtesad S, Kamangar F, Etemadi A, Keshtkar A-A, Hekmatdoost A (2017). Prospective epidemiological research studies in Iran (the PERSIAN Cohort Study): rationale, objectives, and design. Am J Epidemiol.

[18] Pasdar Y, Moradi S, Moludi J, Saiedi S, Moradinazar M, Hamzeh B (2020). Waist-to-height ratio is a better discriminator of cardiovascular disease than other anthropometric indicators in Kurdish adults. Sci Rep.

[19] Alberti KGM, Zimmet P, Shaw J (2005). The metabolic syndrome—a new worldwide definition. Lancet.

[20] Baek T-H, Lee H-Y, Lim N-K, Park H-Y (2015). Gender differences in the association between socioeconomic status and hypertension incidence: the Korean Genome and Epidemiology Study (KoGES). BMC Public Health.

[21] Luo W, Guo Z, Hao C, Yao X, Zhou Z, Wu M (2013). Interaction of current alcohol consumption and abdominal obesity on hypertension risk. Physiol Behav.

[22] Gus M, Fuchs SC, Moreira LB, Moraes RS, Wiehe M, Silva AF (2004). Association between different measurements of obesity and the incidence of hypertension. Am J Hypertens.

[23] Chen Z, Li S, Wang X, Zhang L, Shao L, Tian Y (2020). The incidence of hypertension, overweight, and obesity and relationship with cardiovascular events among middle-aged Chinese: 6 years follow-up results. Zhonghua Xin Xue Guan Bing Za Zhi.

[24] Leggio M, Lombardi M, Caldarone E, Severi P, D'emidio S, Armeni M (2017). The relationship between obesity and hypertension: an updated comprehensive overview on vicious twins. Hypertens Res.

[25] Ruilope LM, Nunes Filho A, Nadruz W, Rosales FR, Verdejo-Paris J (2018). Obesity and hypertension in Latin America: current perspectives. Hipertension y riesgo vascular.

[26] Pasdar Y, Moradi S, Hamzeh B, Najafi F, Nachvak SM, Mostafai R (2019). The validity of resting energy expenditure predictive equations in adults with central obesity: a sub-sample of the RaNCD cohort study. Nutr Health.

[27] Hinnouho G-M, Czernichow S, Dugravot A, Batty GD, Kivimaki M, Singh-Manoux A (2013). Metabolically healthy obesity and risk of mortality: does the definition of metabolic health matter?. Diabetes Care.

[28] Fingeret M, Marques-Vidal P, Vollenweider P (2018). Incidence of type 2 diabetes, hypertension, and dyslipidemia in metabolically healthy obese and non-obese. Nutr Metab Cardiovasc Dis.

[29] Yuan Y, Mu J-J, Chu C, Zheng W-L, Wang Y, Hu J-W (2020). Effect of metabolically healthy obesity on the development of arterial stiffness: a prospective cohort study. Nutr Metab.

[30] Hashimoto Y, Tanaka M, Okada H, Senmaru T, Hamaguchi M, Asano M (2015). Metabolically healthy obesity and risk of incident CKD. Clin J Am Soc Nephrol.

[31] Gilardini L, Zambon A, Soranna D, Croci M, Invitti C (2018). Predictors of the transition from metabolically healthy obesity to unhealthy obesity. Eating Weight Disord Stud Anorex Bulim Obes.

[32] Zhang N, Chen Y, Guo X, Sun G, Dai D, Sun Y (2017). Metabolic abnormalities, but not metabolically healthy obesity, are associated with left ventricular hypertrophy. Heart Lung Circ.

[33] Mongraw-Chaffin M, Foster MC, Anderson CA, Burke GL, Haq N, Kalyani RR (2018). Metabolically healthy obesity, transition to metabolic syndrome, and cardiovascular risk. J Am Coll Cardiol.

[34] Seravalle G, Grassi G (2017). Obesity and hypertension. Pharmacol Res.

[35] Pasdar Y, Moradi S, Abdollahzad H, Hamzeh B, Najafi F, Nachvak SM (2019). Accuracy of waist to hip ratio calculated by bioelectric impedance device in the Ravansar non-communicable disease cohort study. Top Clin Nutr.

[36] Jiang P, Ma D, Wang X, Wang Y, Bi Y, Yang J (2018). Astragaloside IV prevents obesity-associated hypertension by improving pro-inflammatory reaction and leptin resistance. Mol Cell.

[37] Lambert EA, Straznicky NE, Dixon JB, Lambert GW (2015). Should the sympathetic nervous system be a target to improve cardiometabolic risk in obesity?. Am J Physiol Heart Circ Physiol.

[38] Aronow WS (2017). Association of obesity with hypertension. Ann Transl Med..

[39] Cwynar M, Gąsowski J, Gryglewska B, Głuszewska A, Kwater A, Królczyk J (2019). Insulin resistance and renal sodium handling influence arterial stiffness in hypertensive patients with prevailing sodium intake. Am J Hypertens.

[40] Ohashi K, Kihara S, Ouchi N, Kumada M, Fujita K, Hiuge A (2006). Adiponectin replenishment ameliorates obesity-related hypertension. Hypertension.

[41] De Boer MP, Meijer RI, Wijnstok NJ, Jonk AM, Houben AJ, Stehouwer CD (2012). Microvascular dysfunction: a potential mechanism in the pathogenesis of obesity-associated insulin resistance and hypertension. Microcirculation.

[42] Soleimani M (2015). Insulin resistance and hypertension: new insights. Kidney Int.

